# Strangulated Indirect Inguinal Hernia-Containing Bladder: A Case Report

**DOI:** 10.7759/cureus.60108

**Published:** 2024-05-11

**Authors:** Nicholas O Gerard, Tatjana M Mortell, Amin Izadpanah, Cameron W Belding, Steven D Jones

**Affiliations:** 1 Surgery, Tulane University School of Medicine, New Orleans, USA

**Keywords:** groin hernia, inguinal bladder hernia, incarcerated inguinal hernia, strangulated inguinal hernia, bladder hernia

## Abstract

Inguinal hernias involving the bladder are exceedingly rare and pose a diagnostic challenge. Identifying bladder involvement within an inguinal hernia is imperative to avoid iatrogenic bladder injuries and subsequent complications. Here we discuss a case of inguinal bladder herniation and bladder visualization using methylene blue dye intraoperatively. We present a case of a 45-year-old male who presented with a six-hour history of dysuria and a painful non-reducible right-sided groin mass that had previously been reducible for 17 years. Computed tomography demonstrated an irreducible indirect inguinal hernia-containing bladder. Open Lichtenstein repair was performed, and intraoperative methylene blue-dyed saline successfully identified the herniated bladder, preventing iatrogenic bladder injury. This case report demonstrates the importance of preoperative imaging and intraoperative visualization for the prevention of complications in a rare occurrence of a strangulated indirect inguinal hernia-containing bladder.

## Introduction

Inguinal hernias are common with an estimated lifetime risk of 27% in males and 3% in females [1]. There are two types of inguinal hernias, including direct and indirect, which are defined by their relation to the inferior epigastric vessels. Both types of inguinal hernias protrude superior to the inguinal ligament; however, the direct and indirect types protrude medial and lateral to the inferior epigastric vessels respectively [2]. Approximately 500,000 inguinal hernia repairs are performed per year in the United States and nearly 20 million cases per year worldwide [3,4]. Most variations of the repair function reinforce the posterior abdominal wall through the implantation of a mesh (i.e., hernioplasty) or constitute simple repair via anchoring native structures together (i.e., herniorrhaphy) [4]. The concept of hernioplasty was described as early as 1871 by Marcy, while herniorrhaphy was first described in 1887 by Bassini [5,6]. Following advancements in biomaterials, modern hernioplasty was popularized by Lichtenstein in the 1980s, and remains, in some variation, the technique most surgeons use today [7].

Inguinal hernias involving the bladder are rare [8,9]. The first inguinal hernia involving the bladder was reported in 1903 in a man who died following complications of iatrogenic injury of the bladder [10]. Presently, the mortality rate linked to elective groin hernia repair is minimal, only slightly surpassing the general population's baseline risk [11,12]. Although the risks associated with routine groin hernia repair are relatively low, there is a seven-fold increase in mortality for individuals undergoing emergency groin hernia repair [11]. This risk increases even further, reaching a 20-fold increase, when bowel resection becomes necessary in the course of the procedure [11]. Perioperative complications include groin pain, bleeding, seromas, and hydrocele, and chronic complications include hernia recurrence and chronic pain [12,13]. The reported incidence of significant chronic pain ranges from 10-12% and debilitating chronic pain ranges from 0.5-6% [12].

Diagnosis of an inguinal hernia is typically made by identifying a mass in the groin during a physical exam [13]. In some cases, a mass may not be palpable, and symptoms of bowel obstruction may be the only clinical indicators [14]. Diagnostic imaging such as ultrasonography is often performed to confirm the diagnosis of an inguinal hernia with a high degree of sensitivity and specificity [13,14]. Conservative nonoperative measures such as “watchful waiting” can be applied to reducible asymptomatic hernias [4]. However, both incarcerated and strangulated hernias are surgical emergencies occurring in about two in every 1000 cases annually [4]. In this report, we describe the case of a 45-year-old man who presented to the Emergency Department with a chief complaint of a six-hour history of a groin bulge and dysuria, coupled with a one-day history of constipation. Our examination revealed an indirect inguinal hernia-containing bladder requiring emergent surgical intervention.

## Case presentation

The patient was a 45-year-old man who presented to the Emergency Department with a painful right-sided groin mass for a six-hour duration. This was the first instance of this pain which he described as sharp and unrelenting. The patient additionally reported a six-hour history of dysuria and a one-day history of constipation. His past medical history included a previously intermittently reducible right groin mass for the last 17 years and a current BMI of 33.3. He reported he had always been able to push the mass back inside his abdomen or reposition his body in a way to make it disappear. He had no past surgical history, no home medications, and no allergies. Socially, the patient self-reported as a never-smoker and did not drink alcohol, or use illicit drugs.

**Figure 1:**
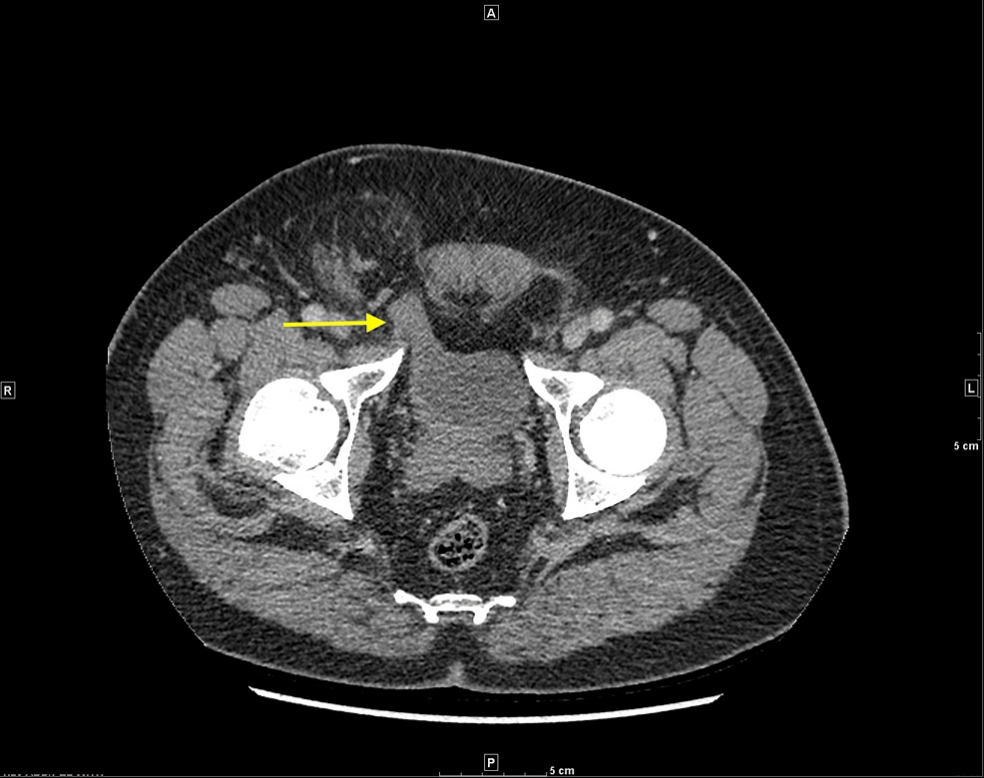
Axial CT image of bladder segment traversing abdominal wall defect into the hernia sac (yellow arrow).

**Figure 2:**
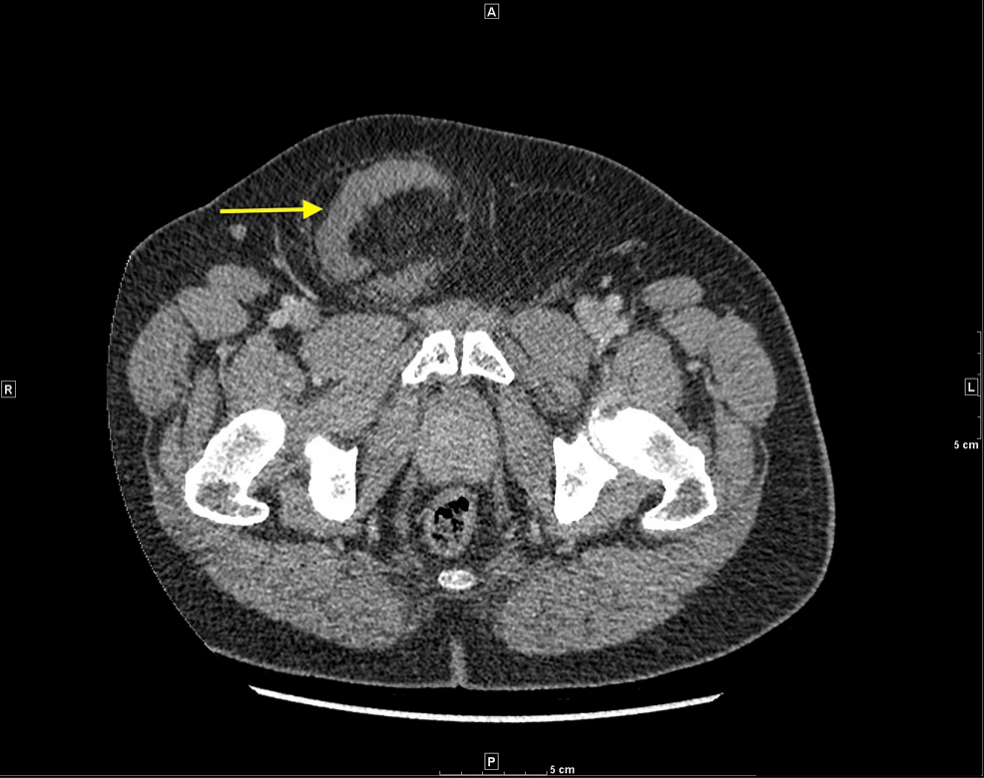
Axial CT image of small bowel segment traversing abdominal wall defect into the hernia sac (yellow arrow).

On physical exam, the patient had a severely tender, soft, non-distended abdomen. In his right inguinal region, he had a non-reducible groin mass measuring 20cm x15cm with mild overlying skin erythema. Once the mass was determined to be non-reducible, we elected to image the mass via computerized tomography (CT) abdomen and pelvis with contrast. CT was chosen over ultrasound given the large size of the mass. CT revealed a right indirect inguinal hernia containing both loops of mildly dilated fluid-filled bowel with fat stranding and the right anterior urinary bladder which contained a small amount of fluid (Figures 1, 2).

**Figure 3:**
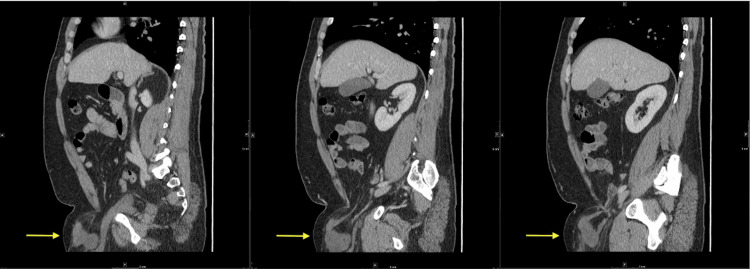
Sagittal CT series of bladder and small bowel segment traversing abdominal wall defect into the hernia sac.

Of note, there was no evidence of strangulation, or upstream dilation on imaging (Figure 3). Given the patient’s acute onset of symptoms and irreducibility of the groin, the patient was taken for an open inguinal hernia repair.

The open Lichtenstein procedure was explained to the patient in detail with assistance from a Spanish-language medical interpreter. All questions were answered, and informed consent was obtained. Under general anesthesia, the patient’s abdomen and groin were prepped and draped standardly. A 10 cm incision overlying the inguinal ligament was carried down through the subcutaneous fat with electrocautery until the hernia sac was identified. The external oblique was identified laterally, and the hernia sac was found to be protruding through a 5 cm defect in the external oblique aponeurosis. The spermatic cord was identified, protected, and retracted away from the operative area.

The hernia sac, found to be approximately 15 cm in diameter, was opened and a small volume of clear fluid was expressed. A segment of the small bowel was found within the hernia sac and appeared dusky, suggesting ischemia. A segment of the pre-peritoneal fat within the hernia was resected and sent for pathology. No perforation was identified, so the bowel was tagged with a Vicryl suture (Ethicon Inc., Cincinnati, OH, USA) and replaced through the hernia defect back into the abdomen. We returned our attention to the remainder of the contents within the hernia sac. We identified a pale, firm, and rubbery structure within the sac which did not appear to be bowel. We confirmed the visualized structure was bladder by introducing methylene blue dyed saline retrograde through the Foley catheter. Rapid filling of the structure with subsequent compressibility confirmed the structure was bladder. We carefully dissected around the hernia sac, and we introduced the bladder into the abdomen. The tagged bowel segment was retrieved, reinspected, and found to be sufficiently reperfused. No bowel resection was indicated, so the Vicryl suture (Ethicon Inc.) was removed, and the bowel was replaced in the abdomen. 

Mesh was placed in the defect, measured, and cut to the appropriate size to cover the defect and allow passage of the spermatic cord. The mesh was fixed to the shelving edge of the inguinal ligament laterally and the external oblique medially. After confirming this spermatic cord moved freely, the incision was closed in layers. The skin was closed with staples and the incision was dressed appropriately. The patient was awoken and was extubated without incident.

Following surgery, patient education included activity restrictions and wound care guidelines. The patient was sent home with over-the-counter multimodal pain control and was seen in the clinic one week post-op for staple removal, and two weeks post-op for follow-up. At both the one-week and two-week post-op follow-up the patient presented without urinary complaints, without evidence of surgical site infection or wound dehiscence on physical exam. The patient was instructed to continue refraining from heavy lifting for another week and to follow up if any symptoms returned. 

## Discussion

In this case, we describe a rare strangulated indirect inguinal hernia-containing bladder in a 45-year-old man. The patient had no prior surgical history, and his only relevant medical history was a previously reducible groin mass for the last 17 years. The primary symptoms of his presentation were pain, dysuria, and constipation. Despite the use of advanced imaging modalities in this case, strangulation was not identified until surgical examination revealed a visibly ischemic portion of the bowel. Thus, if we had not elected to emergently repair this hernia the patient would have likely had a section of his bowel lost to necrosis and possibly a perforated bowel.

The literature ubiquitously cites a statistic originally reported by Levine et al. in 1951, which claims 1-4% of all inguinal hernias involve the bladder [8]. This was established from a 30-patient case series of inguinal hernias involving the bladder. However, there are no recent studies to support this claim. We suggest the incidence of inguinal hernias involving the bladder is exceedingly rare. A recent comprehensive review published in 2018 reported 64 cases of inguinal hernias involving the bladder over a 10-year period [15]. However, we suggest this number is not representative of all occurrences, assuming some cases are not reported in the literature. We would expect more than 64 cases in 10 years, therefore making the 1-4% figure an underestimate of the true incidence of this surgical phenomenon. Further research is warranted to understand the incidence of inguinal hernias involving the bladder; however, it appears the incidence of inguinal hernias involving the bladder is exceedingly rare.

Although the bladder was positively identified on CT imaging prior to surgery, the regional anatomy was distorted in the operating room. This necessitated intraoperative visualization of the anterior bladder wall identified on CT. Intraoperative visualization of the bladder was achieved by introducing normal saline dyed with methylene blue retrograde through the patient's Foley catheter. After retrograde injection, the portion of the hernia sac is assumed to be bladder dilated. Methylene blue was added to the saline to provide pigmentation to enhance visualization and thus assist in identifying possible leaks or defects in the bladder wall. When the tissue was reintroduced into the abdomen, the small distal portion of the bladder visualized became less noticeable. Given the visual and tactile elasticity of the tissue, there was minimal concern for loss of structural integrity of the bladder. Operative specimens were sent to pathology for evaluation and returned with benign findings. Sixth months post-operatively the patient has remained without complaint or complication.

## Conclusions

Inguinal hernias containing bladder are an uncommon surgical pathology. Thorough preoperative evaluation and imaging are critical for diagnosis, appropriate surgical planning, and prevention of iatrogenic bladder injuries. In addition to imaging, the retrograde administration of methylene blue dyed saline through a Foley catheter can also help prevent iatrogenic bladder injuries in such cases.
